# Books and Authors

**Published:** 1912-06

**Authors:** 


### BOOKS AND AUTHORS

**The Care of the Insane and Hospital Management. Charles Whitney Page,** M.D., Supt. Danvers State Hospital, Danvers, Mass. Published by W. M. Leonard, Boston. 155 pages, $1.00.

Tactfully but very candidly, the selection of internes, promotion of assistants, management of nurses, attendants and trustees and various similar delicate topics, are discussed. Non-restraint is the key note of the various scattered sections dealing with the direct care of the patients. The book is scarcely at all technical and should appeal to sociologists, persons engaged in philanthropic work and those unfortunate enough to have an insane relative, as well as to those professionally interested in medicine.

**A Manual of Clinical Chemistry, Microscopy and Bacteriology. Drs. M. Klopstock** and **A. Kowarsky** of Berlin. Authorized, anonymous, translation. Rebman Co., 1123 Broadway, New York. 375 pages, 43 figures and 16 colored plates, $3.00.

This is a well arranged, concise presentation of the subject, designed as a guide to laboratory work, especially by men not too well trained.

**The Treatment of Short Sight. Prof. Dr. J. Hirschberg,** Berlin, translated by **Dr. G. Lindsay Johnson,** Johannesberg. Published by the Rebman Co., New York. 123 pages, 13 illustrations.

While brief and mainly concerned with the operation for the restoration of the eye to its normal length, the author enters somewhat into historic study of myopia, its prevalence, prevention and hygienic treatment. The geometry of myopia is discussed quite technically. The results of operation are tabulated and the author speaks conservatively and with a warning note, against rash operation. This is an epoch-making book.

**The Immediate Care of the Injured.** By **Albert S. Morrow,** M.D., Adjunct Professor of Surgery in the New York Polyclinic. Second Edition, Revised. Octavo of 354 pages, with 242 illustrations. Philadelphia and London: W. B. Saunders Company, 1912. Cloth, $2.50 net.

Part I consists in a rudimentary treatise on Anatomy and Physiology, approximately corresponding to a high school course. Part II describes bandages, dressings, antiseptics, medicaments, etc. Part III deals seriatim with various accidents and emergencies. The author has undertaken a difficult and thankless task, in preparing a work which shall prevent harm from lay ignorance and render the layman a competent but not officious assistant to or fore-runner of the physician and surgeon. It seems to us that Dr. Morrow has performed this task with great skill.

---

**Differential Diagnosis.** Presented through an Analysis of 385 cases. By **Richard C. Cabot,** M.D., Assistant Professor of Clinical Medical, Harvard Medical School. Second Edition. Octavo of 764 pages, illustrated. Philadelphia and London: W. B. Saunders Company, 1912. Cloth, $5.50 net.

Dr. Cabot has reversed the customary method of classification according to disease and very wisely since, if one knows what disease to look under, his differential diagnosis is already made. He has also gone contrary to modern precedent in classifying according to symptoms rather than physical, chemic and other methods of investigation. In so doing, he has followed the order in which cases naturally appear. More than half the book comes under the diagnostic heading of PAIN, variously sub-divided: Fevers, Chills, Haematuria, Dyspnoea, Jaundice and Nervousness follow. The charts suggest Butler's and the arrangement of the work the Case History Series, a comparison which is in no way odious, and in no way derogatory to the originality of the work.

---

**Report of the Commissioner of Education,** for the year ended June 30, 1911. Vol. I. Printed by the Government, Washington, D. C. 1912.

This is a most interesting report on general education but does not enter into statistics of medical education.

---

**New and Non-Official Remedies,** 1912. Published by the A.M.A., Chicago, 1912. 50 cents cloth, 25 cents paper covers. 298 pages including index.

There has been so much discussion as to the general plan and methods of the Council on Pharmacy and Chemistry, and the material contained in this book has been scanned weekly by so many, that we need say little regarding this work. It is of the utmost value, and the Council deserve the highest commendation for their labors. That they should, in every detail, reach the same conclusions as any given critic is manifestly impossible and we trust that all, however interested, will maintain a fair attitude toward both sides in any particular controversy.

---

**The Prevention of Dental Caries, J. Sim Wallace,** D.Sc., M.D., L.D.S. London, published by **The Dental Record,** Alston House, Newman St., W., London, 1912. 70 pages, a few illustrations, 1½ shillings.

We trust we may be pardoned for expressing the price in an American idiom. This is a very thorough and concise treatise, recognizing, of course, the essential role of bacteria in causing caries, and with recognition of Miller's work in dental bacteriology, but taking up practical means of prophylaxis in a highly original manner. The tooth brush, tooth powders and antiseptic washes are considered rather cosmetic than prophylactic of caries. Much stress is laid on the foods favoring and tending to prevent decay, the general principle being that any sticky, soft food, not requiring mastication and liable to remain in contact with the teeth is harmful, while fibrous, detergent masses, requiring mastication tend to prevent decay. Sugar, candy, farinaceous foods, starchy foods with milk or in the form of puddings belong in the former category while meats, vegetables, especially raw, fruits, toast or bread stuffs with a fibrous consistence, fatty foods and liquids, except chocolate, belong in the latter.

---

**Compendium of Diseases of the Skin,** based on an analysis of 30,000 consecutive cases, with a therapeutic formula. **L. Duncan Bulkley,** A.M., M.D., New York. Published by Paul B. Hober, 69 E. 59th St., New York, fifth revised edition of the **Manual of Diseases of the Skin;** cloth, 8 vo., 300 pages, $2.00 postpaid.

When a specialist of long experience and sound education, general as well as in direct preparation for his occupation, bases his work on 30,000 tabulated cases, and condenses his observations into 300 pages of coarse print, the result is obviously practical. To use a homely figure, Dr. Bulkley shows his hand; there is no chance for a bluff. The 30,000 cases are equally divided between private and charity cases and, in each series, the sexes are approximately equal. Hence, conclusions as to sexual and social differences of incidence, course, etc., are reliable. An examination of the hand that Dr. Bulkley has thrown on the table confirms the prima facie favorable opinion. There are about the normal number of exanthematous cases to be expected in dermatologic practice from consultations regarding puzzling and atypic appearances—less than a hundred altogether. Many dermatologists frankly and, therefore, properly, include venereal diseases but in this list, we find no gonorrhoea, only 13 cases of chancroid in 15,000 private cases, though nearly 400 in the charity cases, even syphilis is represented by only 1,000 private and 2,000 charity cases. Similar statements might be made as to various other border-line diseases. Another favorable sign, is the actual rarity of rare diseases; the reader feels certain that there is no manufactured evidence. The same evidence of veracity is shown in the opposite way by the fact that five common diseases—the only ones whose grand totals run above a thousand —comprise 18,700 of the 30,000 cases. These are acne, dermatitis, eczema, psoriasis and syphilis.

---

**Hand-Book of the Anglo-American Medical Association of Berlin.**

This association of English-speaking medical students, in Berlin, has permanent quarters in the Hotel Atlas, 105 Friedrich Strasse. The hand book contains all sorts of information as to lecture courses and all physicians expecting to study in Berlin, would do well to apply for membership. Among the members from November 30, 1910, to January 1, 1912, we note those of C. S. Bentz, J. L. Eckel, and J. C. Roberts of Buffalo, C. E. Rynd of Westfield, R. A. Vose of Ithaca, W. H. Boynton of Brewster.

---

**Text Book of Ophthalmology,** in the form of clinical lectures, **Dr. Paul Roemer,** Greifswald; translated by **Dr. M. L. Foster;** published by the Rebman Co. of New York. 186 illustrations, 13 colored plates, cloth, 3 volumes, $2.50 each.

Volume I, contains 275 large pages, dealing with the methods of examining the anterior segment of the eye, and the diseases of the conjunctiva and cornea, iris and lens. What impresses the reviewer is the sustained effort at clear description and at practical application of scientific facts.

---

**The New Pocket Medical Formulary with an Appendix** containing Formulae and Doses for Hypodermic Medication; Posologic Table; Fractures, Dislocations and Sprains, Ligations of Arteries; Hemorrhages and Wounds; Poisons and Antidotes; Miscellaneous Emergencies; Tables of Differential Diagnosis, Diet Lists for Various Diseases; Formulae for Fluid Foods, etc., by **William Edward Fitch,** M.D., N. Y. City. F. A. Davis Co., Phila. 474 pages, $2.00.

This is a most valuable little companion, well worth the price in spite of the small size. The only unfavorable criticism that occurs to us is that the author, like many others, fails to realize that there is absolutely no use in employing the metric system as a literal translation of an apothecaries' formula. For instance, instead of

| | | |
|---|---|---|
| ℞ | Hydrarg. ammoniat | 3.9 |
| | Cerati simplicis | 31.10 |

which uses inconvenient amounts and affords no idea of strength, one should translate freely and idiomatically, somewhat as follows:

| | | |
|---|---|---|
| ℞ | Hydrarg. ammoniat | 2.50 |
| | Cerati simplicis | 20.00 |

which indicates an 8 per cent. ointment. Similarly, in the liquid formulae which are worse yet and tend too much toward polypharmacy, total bulk should be brought to 100 or some such even quantity, and doses reckoned smoothly as multiples of 5 C.C., the teaspoonful. As in apothecaries' formulae, the teaspoonful is usually estimated at a drachm, which is incorrect, the quantities in the two contrasted formulae will, evidently not correspond either exactly or proportionately.

---

**Immunity. Dr. Julius Citron,** Berlin; translated by **A. L. Garbat,** M.D., New York. Published by P. Blakiston's Son & Co., Philadelphia. 209 pages, 27 illustrations, 2 colored plates, 8 charts. $3.00.

This is a book for study rather than review, as it would be unjust to the author and inadequate for the reader, to select passages at random. The long list of authors indicates the care with which the material has been collated. Very little space is given to abstract discussions of theory yet it would scarcely be correct to say that information of theory is presupposed for, very briefly, and with the attitude of one thoroughly familiar with the practical side of the question, explanations are interpolated so as to afford an intelligent basis for the consideration of experiments and clinical tests.

**Surgery of Deformities of the Face, Dr. John B. Roberts,** Philadelphia. New York, Wm. Wood & Co., 273 pages, 273 illustrations, $3.00

The development of plastic surgery, the removal of gun powder, tattoo marks, etc., disfiguring skin diseases, and the discussion of peculiar cases, methods of grafting, etc., are among the noteworthy portions of the book since we expect the thorough discussion of hare lip, cleft palate, syphilitic deformities, etc. No surgeon can afford to be without this work.

---

**Modern Methods in Nursing.** By **Georgiana J. Sanders,** formerly Superintendent of Nurses at the Massachusetts General Hospital, Boston. 12mo of 881 pages, with 228 illustrations. Philadelphia and London: W. B. Saunders Company, 1912. Cloth, $2.50 net.

A most excellent book. The only crticism that we can offer is to recur to the general problem of how far it is desirable and safe to instruct nurses in methods of diagnosis, especially those involving technicalities of chemistry and bacteriology. We can not help feeling that it is unreasonable to include such subjects in the nursing curriculum and that, unless they can be gone into thoroughly, "a little knowledge is a dangerous thing." In short, it is a question of drawing the line between the nurse and the physician.

